# Postgraduate training in psychiatry in India

**DOI:** 10.4103/0019-5545.69219

**Published:** 2010-01

**Authors:** Shridhar Sharma

**Affiliations:** Department of Psychiatry, National Academy of Medical Sciences and Institute of Human Behavior and Allied Sciences, Delhi

**Keywords:** Indian psychiatric society, medical education, medicine and psychiatry, postgraduate training in psychiatry

## Abstract

This review traces the evolution of modern medical education in India on the one hand and the formation of the Indian Psychiatric Society and the progress of postgraduate psychiatric education on the other hand, all in the context of Indian psychiatry. The topic is covered under the headings standard of psychiatric education, the goals, competencies required, impact of psychiatric disorders, relation of medicine to psychiatry, and the directions for the future of postgraduate psychiatric training.

## INTRODUCTION

Modern medical education in India began in 1822, in a medical school at Calcutta. The medium of instruction was the language of the region, into which English medical books were translated. In the year 1833, Lord William Bentick, the then Governor General of India, appointed a Committee to examine the question of medical education in India. The committee recommended the abolition of the medical school and the establishment of a medical college in its place. Consequently in the year 1835, two medical colleges were started in India, one in Calcutta and the other in Madras. The duration of the medical course was four years, which was extended to five years in 1845.[1] Till 1946, there were only 16 medical colleges and by 1949, the number increased to 29, and in 1958, there were 50 medical colleges. In 2010 the number has increased to 313 with an annual admission of 35,133 undergraduate students.[2]

The Indian Medical Council Act No. XXVII of 1933, was brought into force on 1 November 1933, and the Medical Council of India (MCI) was constituted thereunder on 15 February 1934. From time to time, amendments have been made in this Act. The council has been primarily concerned with undergraduate medical education, but during the last six decades, particularly after the new Medial Council Act of 1956, it empowered the Council to look after Postgraduate Medical Education. According to Section 20(1), the Council may prescribe standards of postgraduate medical education. Consequently, the Postgraduate Medical Education Committee of MCI has made valuable recommendations regarding the nomenclature of postgraduate degrees, the courses and period of studies, examination systems, recognition of training institutes, and postgraduate teachers.

There has been a tremendous growth in medical education since 1935, but more so, during the last three decades. Today there are 313 recognized medical colleges and admit 35133 students every year. As on July 2010, there are 112 Medical Colleges and Postgraduate Institutes, which admit 266 M.D. degree students in Psychiatry each year, besides which, 55 Medical Colleges have training facilities for 124 D.P.M. students. In addition 50 to 60 Postgraduates appear for D.N.B. of the National Board of Examination. It may be important to mention that the first M.D. Psychiatry course was started by Medical College at Patna in 1941 and the first M.D. Candidate was late Prof. L.P. Verma, a Past President of I.P.S. and Past Editor of Indian Journal of Psychiatry and Neurology.

The Indian Psychiatric Society was founded in January 1947 and significantly in the same year the Society appointed a committee on Post-graduate Psychiatry Education. The need for P.G. training in Psychiatry was mentioned by Dr. C.C. Saha during his Presidential address by him in 1962 at Agra.[3] Since then many authors have discussed issues connected with the undergraduate teaching. First, there was a report of the subcommittee on undergraduate teaching in Psychiatry, which was adopted by the Executive Council of the Indian Psychiatric Society on 12 October 1964.[4] Later as a follow up, Directorate-General of Health Services (DGHS) and the World Health Organization (WHO), took another initiative to bring out a monograph on undergraduate training in psychiatry, in India.[5] Eventually the glorious achievements of psychiatric education were highlighted in an editorial of the Indian Psychiatric Society Journal.[6] Since then, intermittently, many articles have appeared in the Indian Journal of Psychiatry, by various authors,[7–16] but only few on postgraduate training in Psychiatry, discussing the Assessment Methods,[17] a survey of postgraduate training centers in India,[18] and another on the role of general hospitals in Postgraduate Teaching.[19] They all made a significant contribution to the advancement of both undergraduate and postgraduate training. Today there are over 103 medical institutions providing postgraduate training in psychiatry. This is a remarkable achievement when compared to the past. From 1947 to 1967 there were only six institutes in India offering postgraduate degrees (MD), and from these centers about 14 psychiatrists qualified as MDs. However, there are no reliable data available on the number of doctors from India trained in the UK and USA, although figures from the Institute of Psychiatry. London, do give an indication of their number. From India alone 101 received training in psychiatry between 1949 and 1966; they formed about 10 percent of the total number of trainees during those years.[20,21]

This trend is gradually changing, and during the last four decades, dramatic developments have taken place regarding the growth of psychiatry. Today it is obvious that like most other specialties, psychiatry is becoming increasingly specialized and fragmented.

## STANDARD OF PSYCHIATRIC EDUCATION

The development of psychiatric education, including its objectives and standards is guided by the Medical Council of India, a statutory body established in 1933. Since 1956 it has had a permanent Committee on Postgraduate Medical Education, whose function it is to formulate rules and curricula of studies and the minimum requirements for teaching centers. It also maintains the quality of teachers and examinations conducted by the universities, so as to bring about uniformity of standards.

In many ways the recommendations of the Council are mandatory and should be fulfilled before an institute/ college is recognized as a Postgraduate Department, and the following areas are essential:

(a) The teaching staff must be adequate and qualified
  (b) The student to teacher ratio must be satisfactory, normally one postgraduate student (MD) per teacher per year-recently this ratio has been changed to two MDs for one teacher from 2010
  (c) The ratio of student to number of patients he handles must be satisfactory
  (d) Each postgraduate teacher must have at least 40 in. patients with out-patient, follow-up and adequate laboratory facilities, and a library for research, in the unit
The selection of MD students must be strictly on merit, and in some institutions credit must be given to their work in rural areas
The duration of a postgraduate degree course must be for a minimum of three years and for the diplomas two years, after one year of compulsory rotatory internship in a recognized medical institution. This period of three years training has to be full-time, and every postgraduate student must be either a full-time resident or a full-time scholar
With regard to the content and method of training, the main purpose must be to expose the student, by graded residency posts, to all branches of clinical psychiatry and also neurology. The student must participate in the care and management of patients, and must be given increasing responsibility as his experience develops. By the time he is in his last year of training, he must be able to diagnose and initiate treatment independently. He has to become conversant with the allied subjects, such as, neuroanatomy, neurophysiology, neurobiochemistry, including electroencephalography, neuroradiology, psychology, and social work, during his training
Organization of the teaching program is a complex task for anyone given this kind of responsibility. It is easy to preach ideals but difficult to practice, and at present our leaders face two formidable tasks: First, to train and provide service to the mental healthcare of 1.2 billion population, and second, to maintain high standards in the scientific field in order to ensure a high quality of teaching and research activity

## GENERAL CONDITIONS

### Duration of training

MD Three years
DPM Two years

The modular approach in teaching is essential. Training must involve learning experiences targeted at the needs of the community and also at exposing the students to community-based activities. A combination of both formative and summative assessment is vital.

### GOAL

To produce competent and knowledgeable specialists and/ or medical teachers and to research in psychiatry.

To achieve this, it is recommended that postgraduate students recognize the health needs of the community, and be able to carry out professional obligations ethically. To master most of the competencies, it is necessary to grasp the speciality required for the needs at the secondary and tertiary levels, and be aware of the contemporary advances in the discipline. To acquire a spirit of scientific inquiry, research methodology and epidemiology are essential. Further it is suggested that they acquire the basic skills in teaching the medical and paramedical professionals.

### General objectives of postgraduate training expected from students at the end of postgraduate training

- Sufficient understanding of the basic sciences relevant to the concerned speciality
- To diagnose and manage the majority of conditions in the speciality
- To plan and advise measures for the prevention and rehabilitation of the mentally sick
- To demonstrate skills in the documentation of individual case records of morbidity and mortality
- To have empathy and a humane approach toward patients and their families
- To have skills for the implementation of a national health program effectively and efficiently
- To organize and supervise healthcare services, demonstrating adequate managerial skills in the clinic/ hospital setting
- To develop a self-directed learning ability, recognize continuing educational needs; select and use the appropriate learning resources
- To develop skills in using educational methods and techniques for teaching of medical students
- To demonstrate being an effective leader of a health team

### Statement of the competences

Similar to every discipline, every postgraduate student in psychiatry shall aim at the development of specific competencies, defined and spelt out in clear terms. Each department shall produce a statement and bring it to notice of the trainees at the start of the program so that he or she can direct the effects toward the attainment of these competencies.

Components of the postgraduate curriculum

The major components of the postgraduate curriculum shall be to include relevant theoretical knowledge and to develop practical and clinical skills and attitudes, including communication skills, training in research methodology, and thesis writing skills.

### The core training curriculum for psychiatry

#### Recommendations

- Basic sciences, in Neuroanatomy, Neurophysiology, Genetics, Neurochemistry, Neuroimaging, Psycho pharmaclogy, Psychology, and Social Sciences, including Statistics and Epidemiology
- Diagnostic assessment of all psychiatric diseases including a basic knowledge of Internal Medicine and Neurology
- Etiopathogenesis
- Therapeutics relevant to all Psychiatric disorders
- Prognosis of various disorders
- Necessary knowledge about prevention and mental health promotion and research methodology

A written curriculum is necessary, as it defines competency and expectation, program evaluation, and helps in the proper assessment of learners.

After the completion of training, the trainees must be knowledgeable about the aspects of those bio-medical, social, and psychological sciences that underpin the practice of clinical psychiatry. In particular, trainees should be able to demonstrate the knowledge of those aspects of neuroanatomy, neurophysiology, neurochemistry, neuropharmacology, and other biological sciences, which are relevant to understanding psychiatric disorders, including those aspects of psychology, sociology, anthropology, and other social sciences, which are relevant to psychiatric disorders. It must also focus on the various biological, psychological, social, and cultural models of the etiology of psychiatric disorders. The theoretical underpinnings of the major treatment modalities for psychiatric disorders, including biological, psychotherapeutic, and social and family interventions are essential.

### Residency: Clinical rotation

A minimum of six months in Neurology and Primary Care, 18 months minimum in General Psychiatry, which includes inpatient, day hospital, rehabilitation, and outpatient services, is necessary. Rotations can include adult, geriatric, child, and adolescent patients. A minimum of six months of complimentary didactic/clinical rotations will be available. It must include three months in consultation and liaison psychiatry and three months in community-based psychiatry.

### General aspects of evaluation

The evaluation of the training process should focus on motivation and empathy. Knowledge in psychiatry, including patient care, communication skills, professionalism, and empathetic development, must be evaluated. Formative and summative assessment should be an integral part of the evaluation process.

### Curriculam: Learning objectives

Appropriate assessment devises should be used for evaluation; for instance, objective structured clinical examination, examination on written or computer-based, multiple-choice questions, chart-stimulated recall oral examination, case logs, and so on. The self-assessment evaluation process should take into consideration the cultural and regional factors.

### Evaluation components

The present evaluation process for the MD degree is not very satisfactory and needs improvement. It should include faculty evaluation by residents, to improve the teaching and development of the faculty. Program evaluation by residents and faculty must be used to improve educational programs. Evaluation of teaching materials and tools must also take place regularly. The role of an individual supervisor in the evaluation process is essential to convey the accumulated evaluation data. Oral and written evaluation must take place twice a year, to determine the level of educational progress and the quality of the curriculum and not just at the end of three years. Patients’ log books must be an integral part of the evaluation process.

### Focus of the evaluation

The evaluation must be compatible and must encompass clinical psychiatry, including clinical assessment and the delivering and monitoring of treatment, and also multiprofessional case management, with ethical professional practice, as also management and leadership issues. The evaluation must also include research, informatics, and knowledge management, and must be demonstrable, culturally sensitive, and deliverable in terms of available local training facilities. Training must have explicit, assessable learning objectives, which must be reviewed and revised as necessary. Similarly evaluation must be rooted in evidencebased practice. There must be feedback from the trainees and the evaluation methods must be modified accordingly.

### Psychiatric disorders

After the completion of their training, the trainees must be knowledgeable about the epidemiology, etiology, psychopathology, clinical features (including complications), and natural history of psychiatric disorders and psychological reactions in both the individual and the caregiver, including concepts of the impairment, disability, and handicap. A sound knowledge of the assessment and care of these conditions is also expected. In particular, trainees must be able to demonstrate the knowledge of the incidence and prevalence of illnesses at different ages and in various populations, as also the history of the evolution of the concepts of psychiatric disorders and principles of treatment. Similarly understanding of the influence of specific factors on the assessment and care of psychiatric disorders, including: Age, intellectual capacity, co-medical illness, gender, culture, spiritual beliefs, and socioeconomic status, are important.

They must also understand the influence of various factors that affect the outcome of treatment and the principles underlying the choice and integration of interventions in psychiatric disorders, including relative cost effectiveness. The principles of legislation, which relate to the practice of psychiatry, with particular emphasis on mental health legislation, including its local application, must also be gathered. The phenomenology of psychiatric disorders, including the definitions of psychiatric symptoms and their significance, psychiatric diagnosis, and the criteria on which these are based, within the framework of one of the widely accepted classification systems such as ICD or DSM, the possible causative or exacerbating factors in psychiatric disorders, the natural history of the disease process in psychiatric disorders, which enables identification of the severity of the disease, the urgency of the need for treatment, the stage of the need for treatment, and the prognosis must be understood.

### Impact of psychiatric disorders

Similarly, after the completion of training, trainees must be knowledgeable about the impact of psychiatric disorders on patients, their families, caregivers, and significant others. They should also have knowledge about the impact on patients with psychiatric disorder and their treatment, including the broader potential impact on multiple areas of the patients’ life and lifestyle. They must also know the particular impact on patients with psychiatric disorder and their treatment, when this treatment involves hospitalization and involuntary treatment. The impact of the psychiatric disorder on the families and caregivers, including an awareness of their needs and their role in the care of a patient is important. Community consequences of psychiatric disorder and implications for the community of policies for the care and treatment of people so affected, is another aspect that trainees need to gain knowledge about, along with understanding the attitudes and responses of the community to psychiatric disorder, including the implications of stigma to the patients, their families, and caregivers.

The trainees must have a knowledge about the appropriate management plans for psychiatric disorders, including (a) Physical and psychological investigations and assessments, (b) Psychotherapeutic techniques, (c) Psychopharmacological and other physical therapies, (d) Situations in which referral to or consultation with colleagues in psychiatry and other disciplines is appropriate, (e) understanding of rehabilitation programs.

### Medicine in relation to psychiatry

After the completion of training, trainees must be knowledgeable about the general medical and common surgical conditions. Higher levels of knowledge, tempered by maturity and experience, are expected in those areas of general medicine that are particularly related to psychiatric practice. In particular, trainees must be able to demonstrate knowledge of the presentation, investigation, diagnosis, and treatment of medical conditions, particularly in those areas related to psychiatric practice. Their knowledge of further investigations that are necessary to confirm or reject diagnostic hypotheses, to aid the patient’s management, the basic principles involved in the management of significant medical illnesses, the interaction between medical and psychiatric disorders, the psychosocial and cultural aspects of medical illness and its significance to patients and their families, with regard to both the illness and its treatment, are desirable.

## LONG CASE HISTORY

The long case history is an essential component of the final examination. Time – one hour, personal style, format – case history, identifying data, How the patient presented his problem – which led to his presentation to the clinic, history must be sufficient, with details – which help to give meaning and substance to the diagnostic formulation and help to arrive at a diagnosis, and the context in which therapy would be affected, including a rehabilitation plan. It must also include physical examination – neurological, cardiovascular system, respiratory system and eye-fundus.

### Short case

1. Title – 15 minutes
2. Personal Style
3. Format must include
  - Mental status examination
  - Neurological examination if required
  - Brief history – relevant
  - Symptoms
  - Signs
  - Diagnostic formulation with tentative diagnosis, with justification
  - Treatment planning

### Spot

Viva must include

- Pharmacotherapy
- Behavior therapy
- Psychotherapy
- Forensic psychiatry
- History of psychiatry
- Clinical syndromes
- Recent research
- Any other aspects

Spot test must include

- Psychological tests
- EEG
- X-rays + MRI + CAT

## CONCLUSION

Psychiatrists have played a prominent role in shaping the mental health program and providing mental health care to patients in every country. It is true for India too. However, their influence is in part a byproduct of their own professional preparation. The global information about the quality and quantity of training of psychiatrists is largely unavailable. Similarly, there is a wide variation in the training programs, in spite of MCI regulations and guidelines. There is a constant need for retrospective introspection.[22] How satisfactory is the training in the changing social, economic, and technological environment, is an area that needs constant evaluation and improvement. Similarly, the mechanism to regularly incorporate the latest knowledge in the curriculum and the data on such matters is not easily available in our country.

Recently, WHO in collaboration with the World Psychiatric Association (WPA) has brought out an interesting publication ‘Atlas: Psychiatric Education and Training Across the World 2005’[23]. This is a good attempt to answer some of the relevant questions. It is a joint publication of the WHO and the WPA. At the global level, the Atlas provides an overview of the situation and brings out the existing regional variation. It also reveals a general deficiency and a marked variability in training across the world. The report provides some useful data regarding the availability of mental health resources. The major limitations of the study are the low response rates from countries. The responses have been received from 73 countries out of the 192 countries in the world. Even among those who responded, many of the key contacts are those who are not directly involved with the policies and implementation of the Medical Education Policy in their countries. This has given rise to some gross inaccuracies and faulty conclusions. To fulfill this complex and difficult mission, it is essential to include persons who are responsible for medical education and training in their country and are knowledgeable about the laws and policies in the field of medical education and health services. The following parameters are suggested as useful guidelines for future studies:

1. The structure and requirements of the healthcare delivery system in each country
2. The role of legal and other accreditation bodies such as the MCI, established under the law of the land, which define and set standards of postgraduate medical education in the universities and medical institutions, which maintain and implement standards and impart training

It needs to be emphasized that the goal of postgraduate training in all specialties, including the field of psychiatry, is to produce competent and knowledgeable specialists and teachers, who can meet the needs of the community, carry out professional obligations ethically, be aware of contemporary advances in the discipline, and have some foundation in the principles of research methodology.

There are some established systems in the education system that are applicable in the field of postgraduate training in medicine, including psychiatry, which can be usefully incorporated to help in this direction, namely, (a) uniform admission policies, (b) some uniformity in the content of the training program, (c) the organization of the curriculum, which can be evaluated (d) the outline of the training program including the standard methods of instruction, (e) objective assessment methods, (f) to develop acceptable guidelines with regard to the relationship between the institution/College/University and the external, national or Today, specialists licensed in one country rarely have extended practice privileges in another country. In some countries, the license for specialists is even restricted for practice in only some provinces of the country. The implications are obvious–the assessment method must be harmonized within the country and some international standards need to be developed–WHO is an established body under the U.N. Organization. Accordingly, WHO is a body that can take the necessary steps to meet this goal. It is not an easy path, but needs patience and sustained efforts to meet the challenge.

The WHO, whose primary mission is that of directing and coordinating International Health Work, must take vigorous steps to develop the standards of medical education at the undergraduate and postgraduate levels and strengthen the accreditation process. This will certainly improve the quality of health care.
